# Multi-wavelength growth of nanosecond laser-induced surface damage on fused silica gratings

**DOI:** 10.1038/s41598-017-18957-9

**Published:** 2018-01-17

**Authors:** Maxime Chambonneau, Laurent Lamaignère

**Affiliations:** grid.457346.7 CEA, CESTA, F-33116 Le Barp, France

## Abstract

The nanosecond laser-induced damage growth phenomenon on the exit surface of fused silica grating is investigated at 1064 nm and 355 nm separately and also simultaneously. Experiments are first carried out on damage sites on a plane fused silica sample showing two different morphologies, and a damage type is selected for ensuring the repeatability of the subsequent tests. Comparing the mono-wavelength growth results on a grating and a plane fused silica sample, the periodic surface structure is found to be an aggravating factor for damage growth. This is highly supported by calculations of the enhancement of the optical electric field intensity thanks to Finite-Difference Time-Domain simulations. Finally, the mono-wavelength results enable us to quantify a coupling occurring in the multi-wavelength configuration, which could originate from the heating of the plasma (more likely produced in the ultraviolet) preferentially by the infrared pulse. This study provides interesting results about the involvement of the surface topography in damage growth, and paves the way towards the comprehension of this phenomenon at high-energy nanosecond laser facilities where fused silica gratings are simultaneously irradiated at several wavelengths.

## Introduction

Although laser-induced damage (LID) phenomenon is almost as old as the invention of the laser^1^, efforts are still required for fully understanding this issue. Researches on this topic have been particularly galvanized in the nanosecond regime thanks to inertial fusion class lasers such as the Laser MégaJoule (LMJ, in France)^2,3^, or the National Ignition Facility (NIF, in the USA)^4^. On such facilities LID shows harmful consequences on the different optical components, including a loss of transmission, a degradation of the output wavefront, and a risk of damaging the downstream components. In the particular case of fused silica optics, LID sites initiated by parallel beams are preferentially located on the exit surface of the components^5,6^, and their size may exponentially increase after successive irradiations^7^. This latter phenomenon known as “LID growth” mainly originates from the absorption of the laser flux by subsurface cracks under a mechanically modified material that may reignite the damage process^8^, leading to an expansion of these cracks as well as a material ejection induced by shock waves^9^. In order to predict the lifetime of the fused silica optics at the LMJ and the NIF, several works have characterized the LID growth at ~1064 nm (1ω)^10–12^, as well as at ~355 nm (3ω)^13–16^, namely the wavelengths mainly employed on these facilities. However, these mono-wavelength studies are not able to predict the growth behaviour in a multi-wavelength configuration [i.e., by combining infrared (IR) and ultraviolet (UV) pulses], inherent to the irradiation conditions of specific components. In order to solve this issue, a coupling between the wavelengths that occurs during LID growth in this latter configuration has been reported in several articles^17–19^. Although this coupling has been characterized for plane surfaces, it may not necessarily be directly applicable for rough or periodically structured ones. Indeed, the random roughness of a surface is known to be an aggravating factor for the initiation of LID^20^, that is generally explained by a local enhancement of the optical electric field^21,22^. For quantifying the influence of a periodic surface structure on LID initiation, experimental works are often carried out on gratings (i.e., an arrangement of periodic pillars)^23^, where the electric field distribution can be determined more easily^24^. Nevertheless, to the best of our knowledge, there are no similar works for LID growth on the surface of a grating so far, neither in a mono- nor in a multi-wavelength configuration. At the LMJ, transmission gratings in fused silica are employed for focusing the beam at 3ω on the target, and also for separating the beams at 1ω and 527 nm (2ω) which are unconverted by frequency conversion crystals^25^. No growing damage site is detected neither on the exit surface nor on the entrance surface of these components at this facility. These performances are partly due to the pillars composing the gratings which are located on the entrance surface implying that the exit surface is a highly resistant plane one, and also that the potential damage sites on the entrance surface would grow linearly rather than exponentially^7^. However, studying mono- and multi-wavelength LID growth with the pillars located on the exit surface of the grating would show two interesting applications. Firstly, this study would validate the choice of the aforementioned configuration for the gratings at the LMJ, selected for increasing the lifetime of the components. Secondly, in a fundamental point of view, it would enable to quantify the impact of the surface topography on LID growth by comparison with a plane polished fused silica sample, and shed light on the role of the surface state on this phenomenon.

In the present study, the LID growth behaviour on the exit surface of a fused silica grating is determined at 1ω and 3ω separately and also simultaneously, by measuring both the probability of growth and also growth coefficient. Prior to present the results on gratings, the influence of the initial damage morphology on the growth results is investigated for a plane silica sample. A significant difference is observed between two different damage morphologies, leading us to carry out growth experiments only on damage sites initiated at 3ω with a similar size for avoiding disparity in the results. Then, the influence of the surface topography on LID growth is evaluated in the mono-wavelength cases by comparing the experimental results on a grating with the ones that we have previously established on a plane fused silica sample. The periodic surface structure is found to be an aggravating factor of LID growth, which is supported by calculations of the electric field enhancement. Finally, the mono-wavelength results enable us to observe and characterize a coupling between the IR and the UV pulses on the surface of the grating in the multi-wavelength configuration. The physical mechanisms that may be involved in the experiments are discussed.

## Results and Discussion

### Dependence of the growth behaviour on the initial damage morphology

**Figure 1:**
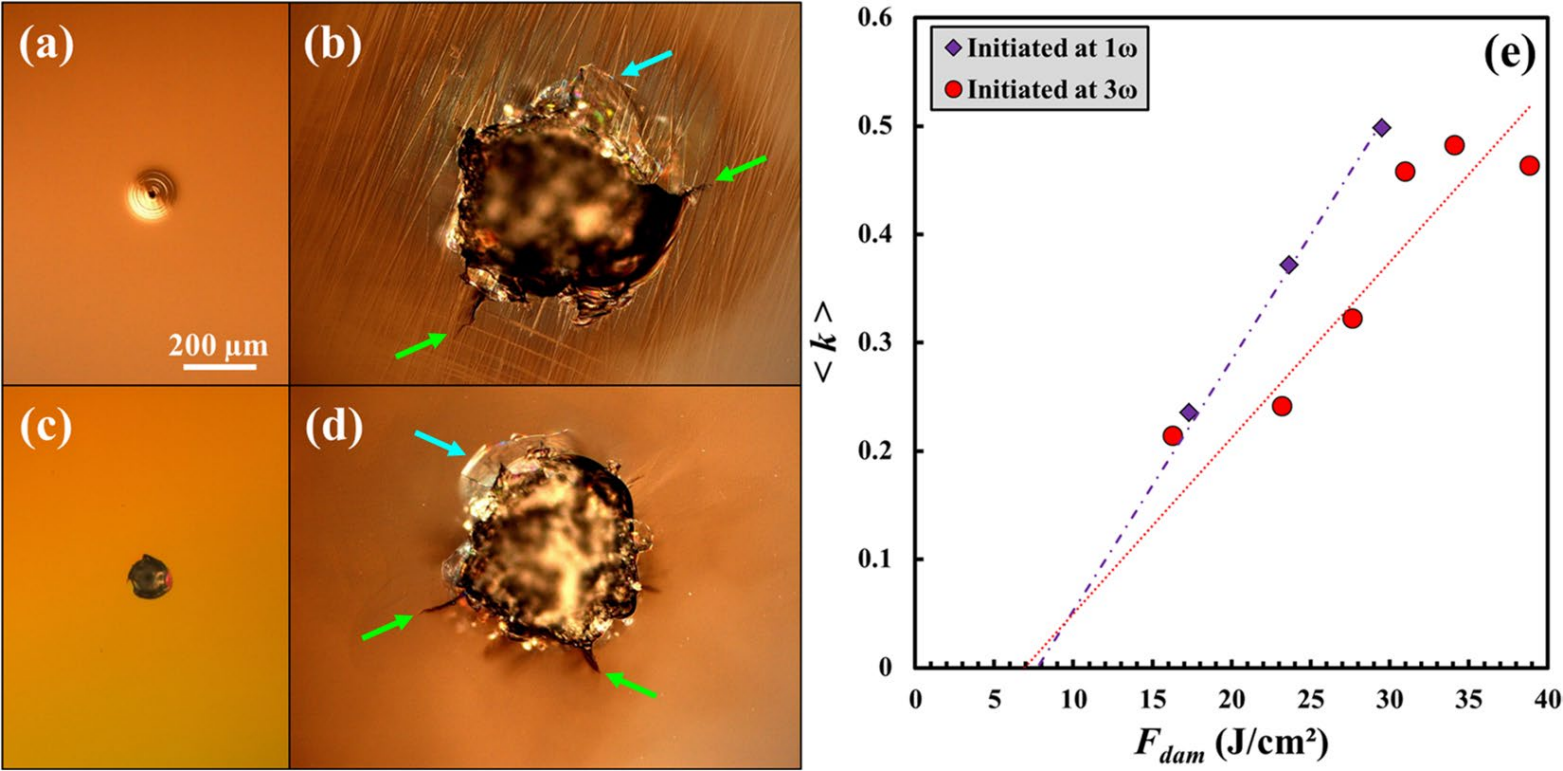
(**a**) Nomarski Interference Contrast micrograph of a laser-induced damage initiated on a plane sample at 1ω, and (**b**) the corresponding site after a growth sequence of 15 pulses of 27 J/cm^2^ at 1ω. (**c**) Nomarski Interference Contrast micrograph of a laser-induced damage initiated at 3ω, and (**d**) the corresponding site after a growth sequence of 15 pulses of 27 J/cm^2^ at 1ω. The spatial scale applies to all images. The green and blue arrows indicate the subsurface cracks and the chips, respectively. (**e**) Evolution of the average growth coefficient $\langle k\rangle$ as a function of the corrected fluence *F*_*dam*_ at 1ω for damage sites initiated at 1ω (purple squares) and 3ω (red circles). The dotted lines stand for linear fits of the whole set of experimental data.

Prior to determine the growth behaviour on grating in the mono- and the multi-wavelength configurations, we evaluate the impact of the initial damage morphology on the growth results on the exit surface of a plane fused silica sample. To do this, we benefit from the longitudinal mode beating inside the laser cavity. Indeed, since the employed pulses show multiple longitudinal modes and thus strong temporal modulations, the morphology of the damage sites initiated at 1ω on the exit surface of fused silica systematically exhibits a typical ring structure Fig. 1(a), originating from the expansion of a plasma driven by the intensity spikes^26^. The morphology of the damage sites initiated at 3ω Fig. 1(c) is much different and often referred to as “pansy”^27^, which consists in a molten core surrounded by a fractured periphery^27,28^. This wavelength-dependence of the damage morphology is due to the ionization threshold of neutral species in air, which is easily reached in the IR and not in the UV. One should also mention the different subsurface crack distributions for both morphologies since they are expected to play a major role in the growth phenomenon^8^. Indeed, when initiated at 3ω, cracks are homogeneously distributed under molten and fractured core^19^, while they follow an annular feature as the surface does when initiated at 1ω^26^. On both sites exhibiting a similar size (~100 µm in diameter) displayed in Fig. 1(a) and (c), an identical damage growth sequence consisting in 15 pulses of 27 J/cm^2^ at 1ω has been applied. The corresponding sites after this sequence are shown in Fig. 1(b) and (d) for the damage sites initiated at 1ω and 3ω, respectively. One can note several resembling features on both damage sites after the growth session. Indeed, for both sites, a molten core (in dark) is surrounded by large cracks and chips indicated by the green and blue arrows, respectively. This emphasizes the importance of thermal and hydrodynamic phenomena, independently of the initial morphology. However, scratches are systematically observed around grown damage sites initiated at 1ω. Due to their random orientation, these likely originate from the polishing processes, and were undetectable with optical microscopy prior to the damage growth experiments because of their location under a densified layer (often referred to as Beilby layer^29^). These scratches may have been revealed by the activation of the surface by the electrons of the plasma formed in the vicinity of the subsurface cracks, followed by a slight ablation (of a few µm) by the 1ω pulse^26^. This process is consistent with the colour gradient around the central crater in Fig. 1(c) [visible thanks to the employed Nomarski Interference Contrast (NIC) technique] which indicates a funnel-shaped area around it. Although such scenario could also be true for the sites initiated at 3ω, no scratches are revealed after the same growth sequence around these sites. Additionally, the only colour change in the periphery of the crater is not ring-shaped and may be caused by the residual stress inside the material^30^. We explain these differences by the initial distribution of the subsurface cracks which are much more localized for pansy damage sites than for ring-patterned ones. Thereby, collective effects may take place for sites initiated at 3ω, resulting in stronger ablation processes. The damage initiation and growth both at 1ω may thus find applications for revealing the polishing-induced scratches. It is worth noting that although the initial damage size is the same in Fig. 1(a) and (c), the damage initiated at 1ω is larger than the one initiated at 3ω after the same growth session [by comparing Fig. 1(b) and (d)], suggesting that ring-patterned sites lead to a more important increase in the damage size.

To explore this assumption, we have carried out a statistical study on the growth of damage sites with the two aforementioned morphologies. Due to the exponential behaviour of the damage area on a pulse-to-pulse basis, the growth coefficient between two laser pulses is calculated as:

1 $${k}_{n}=\,\mathrm{ln}(\frac{{A}_{n+1}}{{A}_{n}})$$

where *A*_*n*_ and *A*_*n*+1_ are the damage area after the pulse *n* and *n* + 1, respectively. During the growth sequence, each laser pulse of equivalent area *S*_*eq*_ (defined at 1/e) with a measured maximum fluence *F*_*max*_ is associated with the average fluence *F*_*dam*_ corrected from the area of the damage site *S*_*dam*_, evaluated as^19^:

2 $${F}_{dam}={F}_{g}\frac{{S}_{g}}{{S}_{dam}}(1-{e}^{-\frac{{S}_{dam}}{{S}_{g}}})+{F}_{w}\frac{{S}_{w}}{{S}_{dam}}(1-{e}^{-\frac{{S}_{dam}}{{S}_{w}}})$$

where ${F}_{g}={F}_{max}|\frac{{S}_{eq}-{S}_{w}}{{S}_{g}-{S}_{w}}|$, and ${F}_{w}={F}_{max}-{F}_{g}$ are the fluence of the Gaussian peak (with area *S*_*g*_) and the wings (with area *S*_*w*_), respectively. Previously, we have shown that this correction was essential when working with beams that are comparable in size to the damage sites^19^. The evolution of the average growth coefficient $\langle k\rangle$ is displayed as a function of the corrected fluence ${F}_{dam}$ in Fig. 1(e) for damage initiated at 1ω and 3ω. For each initial morphology, the whole set of data [i.e., all the ${k}_{n}=f({F}_{dam})$ measurements] is parameterized by:

3 $$\langle k\rangle =C({F}_{dam}-{F}_{th})$$

where *C* is the rate of increase in the growth coefficient, and *F*_*th*_ is the fluence threshold for growth. This latter parameter is almost the same for both damage types (7.7 and 6.9 J/cm^2^ for the sites initiated at 1ω and 3ω, respectively), which is consistent with the previously discussed collective effects that are less important when the fluence is low. However, for higher fluences (>20 J/cm^2^), due to the localized character of the subsurface cracks of pansy damage, $\langle k\rangle$ is lower for the damage sites initiated at 3ω ($C=1.6\times {10}^{-2}$ cm^2^/J) than for the ones initiated at 1ω ($C=2.3\times {10}^{-2}$ cm^2^/J).

In the following growth experiments, we choose to work on single-pit, 60 to 120 µm in diameter, pansy damage sites initiated at 3ω for two reasons. Firstly, the number and the size of the rings initiated at 1ω differ from one site to another even under the same experimental conditions^26^, which could lead to non-repeatable results. Secondly, growth experiments will be carried out at an angle of incidence of 25°, implying that the ring pattern initiated at normal incidence at 1ω is turned into an egg one which may induce an inhomogeneous distribution of the subsurface cracks that could complicate the interpretation of the results^31^.

### Impact of the periodic surface structure on mono-wavelength damage growth

**Figure 2:**
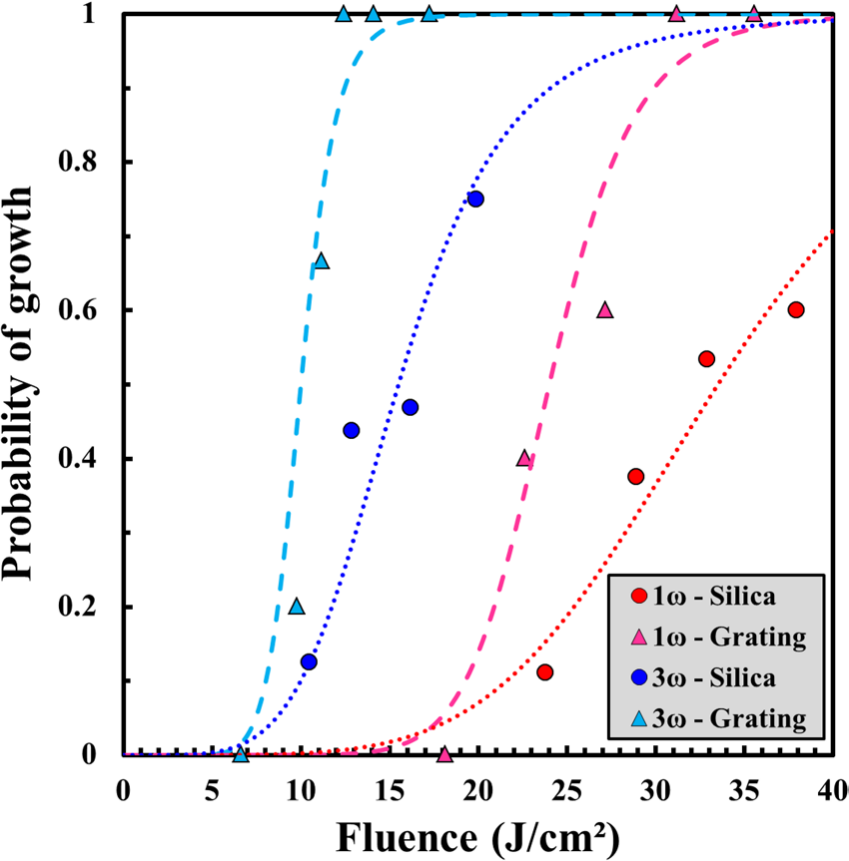
Evolution of the probability of growth as a function of the laser fluence on the plane sample (circles) and the grating (triangles) at 1ω and 3ω. The dotted and dashed sigmoid curves are issued from Eq. (4) with the parameters reported in Table 1.

In order to evaluate the influence of a periodic structure on LID growth in the mono-wavelength configurations, results on the exit surface of a grating are compared to the ones obtained on the plane exit surface of a fused silica sample. These latter issued from Ref.^19^ only serve as a reference for establishing the impact of the topography on the damage growth. For a better characterization of the growth behaviour, two aspects are systematically distinguished: (i) the starting of the growth, evaluated by the probability of growth, and (ii) the evolution of the area of growing damage sites on a pulse-to-pulse basis determined with the growth coefficient. We first determine the probability of growth *P* (i.e., the ratio between the number of growing damage sites and the total tested ones at a given fluence) in the mono-wavelength configurations on the grating and the fused silica sample in Fig. 2. The tested grating is an arrangement of periodic pillars of 700 nm height and 213 nm full width at half maximum regularly spaced with a period of 400 nm, etched on the exit surface of the same substrate as the 10 mm thick fused silica superpolished sample. The data obtained in the four configurations are parameterized by sigmoid curves reading^32^:

4 $$P(F)=1-\frac{1}{1+{(\frac{F}{{F}^{(50 \% )}})}^{p}}$$

where ${F}^{(50 \% )}$ is the fluence for which the probability of growth is equal to 50%, and the exponent *p* determines the shape of the sigmoid curve. The values of these two parameters obtained in the four configurations in Fig. 2 are reported in Table 1. At each wavelength, the probability *P* is higher on the grating than on the plane sample for a same fluence. Moreover, the fluence range for which the probability passes from low (~0) to high (~1) is wider on the plane sample, as emphasized by the exponent *p* in the sigmoid curves which is twice as great for the grating. One should also note that this exponent only depends on the tested component, independently of the employed wavelength. The important difference in the ${F}^{(50 \% )}$ values between the two samples at both wavelengths clearly demonstrates that the damage growth phenomenon is favoured by the periodic surface structure. Therefore, the starting of growth not only depends on the subsurface cracks as shown in Refs^8,33^, but also on the surface topography.

**Table 1:** Parameters of the sigmoid curves employed for parameterizing the probability of growth results in Fig. 2 according to Eq. (4).

Wavelength – Topography	1ω – Plane	1ω – Grating	3ω – Plane	3ω – Grating
*F*^(50%)^ (J/cm^2^)	33.5	24.0	15.5	10.0
*p*	5	10	5	10

**Figure 3:**
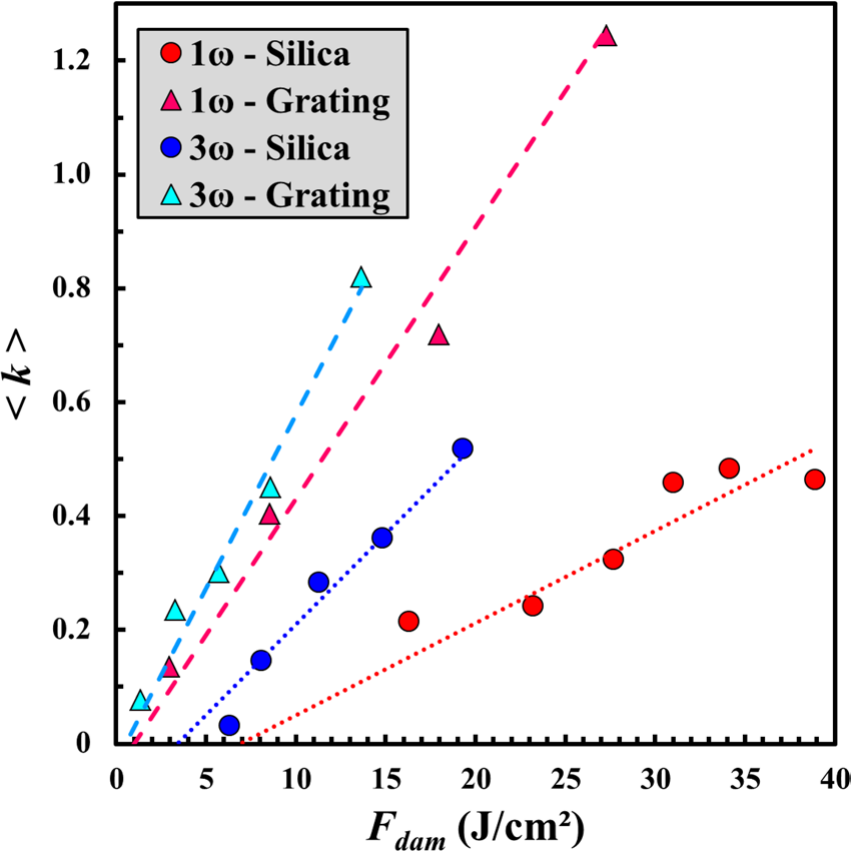
Evolution of the average growth coefficient $\langle k\rangle$ as a function of the corrected fluence *F*_*dam*_ on the plane sample (circles) and the grating (triangles) at 1ω and 3ω. The dotted and dashed curves are issued from Eq. (3) with the parameters reported in Table 2.

Then we investigate if the differences between the two topographies that are exhibited for the starting of growth (i.e., the very first irradiations) are also observed during a growth sequence. The average growth coefficient $\langle k\rangle$ is reported in Fig. 3 as a function of the corrected fluence *F*_*dam*_ defined in Eq. (2) on plane sample and the grating at 1ω and 3ω. Following the same methodology as in Fig. 1(e), $\langle k\rangle$ is shown by the points, and the whole sets of data are parameterized according to Eq. (3). The rate of increase in the growth coefficient *C* as well as the fluence threshold for growth *F*_*th*_ in each configuration are reported in Table 2. On both components, the pulses at 3ω provoke a more significant growth than at 1ω. Moreover, for identical wavelength and fluence, the growth coefficient is much more important for the grating than for silica, as emphasized by the rate *C* which is about three times as great on the grating as on the plane sample at 1ω, and twice as great at 3ω. Interestingly, the difference in $\langle k\rangle$ between the two components at a same wavelength is more significant for the highest *F*_*dam*_ values, mainly corresponding to the smallest damage areas according to Eq. (2). This contrast may be explained by the fact that during the first irradiations of a growth sequence, the small sites (a few tens of µm deep^19^) exhibit short cracks close to the surface which are few in number^34^, so they are strongly affected by the electric field enhancement on the surface induced by the pillars. By contrast, at the end of a growth sequence, large sites showing numerous long cracks deep inside the bulk are less affected by the surrounding surface state. In this case, the damage growth is thus mainly driven by these cracks (few hundreds of µm long), as shown in Refs^8,33^.

**Table 2:** Parameters of the linear curves employed for parameterizing the growth coefficient results in Fig. 3 according to Eq. (3).

Wavelength – Topography	1ω – Plane	1ω – Grating	3ω – Plane	3ω – Grating
*C* (cm^2^/J)	1.6 × 10^−2^	4.8 × 10^−2^	3.2 × 10^−2^	6.1 × 10^−2^
*F*_*th*_ (J/cm^2^)	6.9	1.0	3.4	0.5

**Figure 4:**
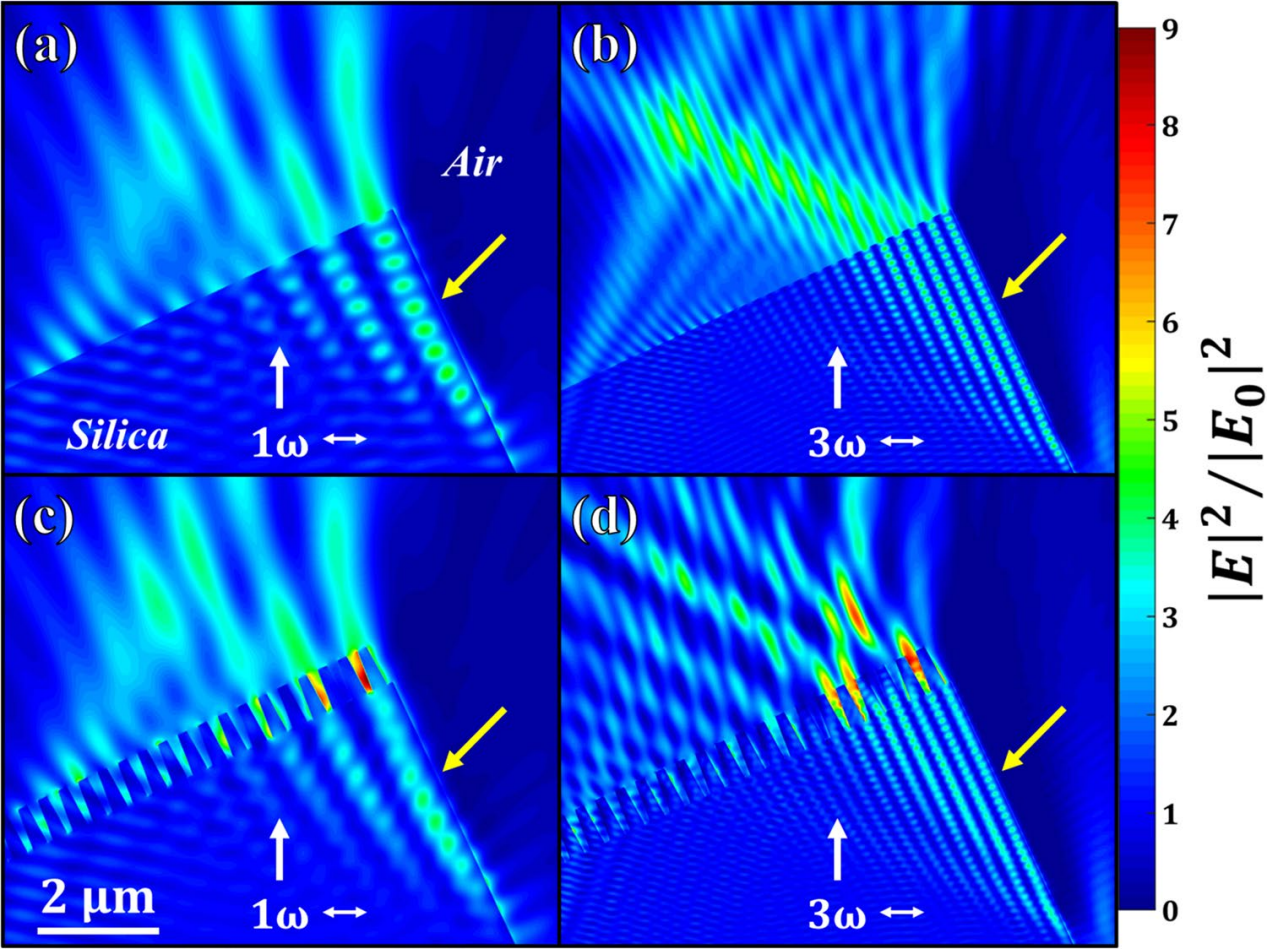
Comparison between the optical electric field intensity distribution ${|E|}^{2}/{|{E}_{0}|}^{2}$ on the surface of plane sample [(**a**) and (**b**)] and the surface of the tested grating [(**c**) and (**d**)] at 1ω [(**a**) and (**c**)] and 3ω [(**b**) and (**d**)] in the vicinity of a laser-induced damage site whose wall is indicated by the yellow arrow. The white single-ended and double-ended arrows indicate the direction of propagation of the plane wave and the laser beam polarization, respectively.

In order to explain the results in Figs 2 and 3, two-dimensional Finite-Difference Time-Domain (FDTD) simulations have been performed. These calculations displayed in Fig. 4 enable us to determine the optical electric field intensity distribution ${|E|}^{2}/{|{E}_{0}|}^{2}$ in the vicinity of a damage site. Since the grating structure is much smaller than the size of the damage sites tested for growth, the wall of the considered damage (indicated by a yellow arrow) is locally perpendicular to the exit surface of the sample at the scale of the pillars. Four configurations have been examined including two different surface states (plane sample and grating), and two wavelengths (1ω and 3ω). The laser beam polarization, the angle of incidence of the sample, and the structure of the grating are the same as in the experiments. These calculations reveal two major results. First, in Fig. 4(c) and (d), the intensity in the air region between the pillars located in the vicinity of the damage site is significantly enhanced (≈9). Since in the plane case this intensity enhancement is more than half as much, the damage process (and thus the propensity of damage sites to grow) is more easily reignited on the grating. This is particularly consistent with the experimental results in Figs 2 and 3 where both the probability of growth and the growth coefficient are higher on the grating than on the plane sample. The second important result is that the maximum electric field intensity inside the pillars is more enhanced at 3ω (≈4.6) than at 1ω (≈2.5). This originates from the grating period (400 nm) which is on the one hand much smaller than the IR wavelength (1064 nm) and on the other hand comparable to the UV one (355 nm). Thereby, the grating irradiated at 1ω is subwavelength and almost behaves like a homogeneous material. Nevertheless, as previously discussed, the intensity at the air/silica interface in Fig. 4(c) is strongly enhanced compared to the case of a plane surface. The more important intensity enhancement at 3ω in addition to the higher photon energy at this wavelength explains the more significant damage growth on the grating in the UV than in the IR in Figs 2 and 3.

Although the calculations of electric field intensity enhancement displayed in Fig. 4 give valuable information for qualitatively interpreting why the LID growth phenomenon is more important on the surface of the grating, a more complex model would be needed to fully explain the growth threshold reduction of a factor ~7 experimentally observed between the two topographies. The two main ionization mechanisms governing the interaction of a nanosecond laser pulse and fused silica are multiphoton ionization and electron avalanche. The nonlinearity associated with both these processes may partly explain why the calculated factors in intensity do not match the reductions in the damage growth thresholds between the plane surface and the grating. This difference may also originate from the grating manufacturing process involving photoresist coating, chemical treatment, and ion etching^25^. The contamination by chemical species on the surface can act as precursor defects able to reignite the damage process. Moreover, the ion etching locally modifies the material mechanically, leading to nanoscale indentations that may play a significant role in the damage growth threshold reduction^35,36^.

### Damage growth on grating in the multi-wavelength configuration

**Figure 5:**
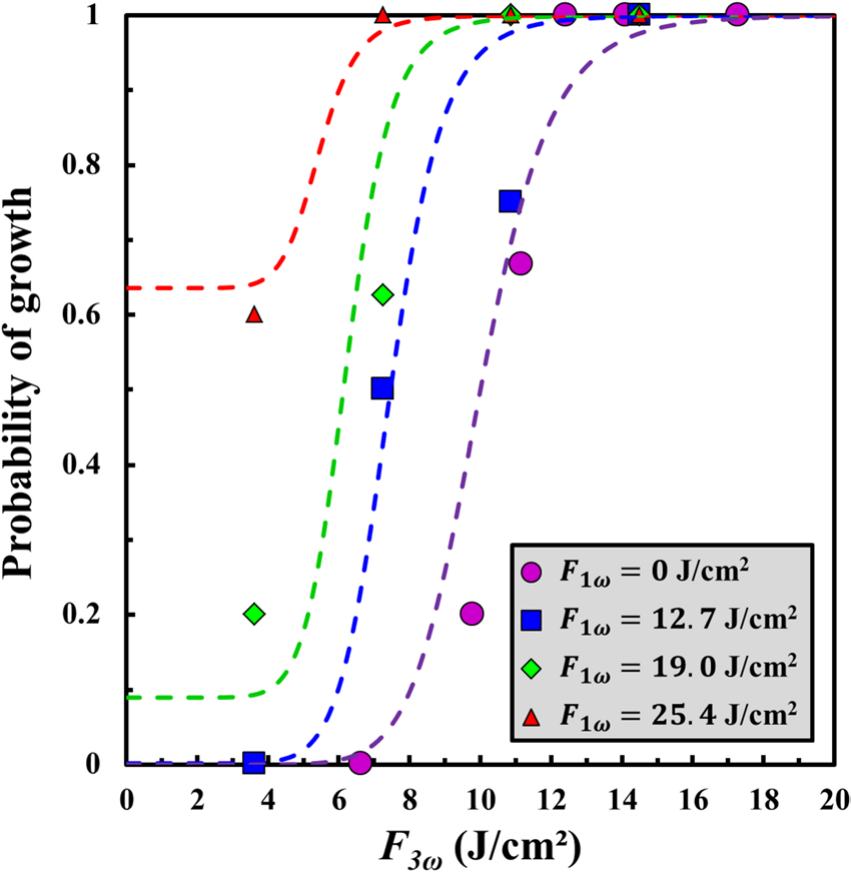
Evolution of the probability of growth on the exit surface of the grating as a function of the laser fluence at 3ω (${F}_{3{\rm{\omega }}}$) for several fluences at 1ω (${F}_{1{\rm{\omega }}}$). The experimental data are compared to the parameterizations issued from Eq. (5) at the same fluences at 1ω (dashed curves in the same colours) with the parameters established in the mono-wavelength configurations reported in Table 1.

On the basis of the results obtained in the mono-wavelength cases, laser-induced damage growth is investigated following the same approach in the multi-wavelength configuration. We have first studied the influence of the combination of 1ω and 3ω pulses on the probability of growth on the same grating as the one tested in the previous section. The corresponding results are displayed in Fig. 5 as a function of the fluence at 3ω (${F}_{3{\rm{\omega }}}$) with three added fluences at 1ω (${F}_{1{\rm{\omega }}}$). These cases correspond to (i) ${F}_{1{\rm{\omega }}}=12.7$ J/cm^2^, where $P < 0.01$ at 1ω only, (ii) ${F}_{1{\rm{\omega }}}=19.0$ J/cm^2^, where $P\approx 0.09$ at 1ω only, and (iii) ${F}_{1{\rm{\omega }}}=25.4$ J/cm^2^, where $P\approx 0.65$ at 1ω only. Figure 5 shows that the addition of a fluence at 1ω to another one at 3ω results in a shift of the sigmoid curves towards the lowest ${F}_{3{\rm{\omega }}}$ values. Hence, for similar fluences at 3ω, the probability of growth increases with respect to ${F}_{1{\rm{\omega }}}$. For instance at ${F}_{3{\rm{\omega }}}=7$ J/cm^2^ where $P < 0.01$ at 3ω only, this probability is 0.63, and ~1 for 19.0, and 25.4 J/cm^2^, respectively. More interestingly, in the case where ${F}_{1{\rm{\omega }}}=12.7$ J/cm^2^ and ${F}_{3{\rm{\omega }}}=7$ J/cm^2^, it is worth noting that although these two fluences are not sufficient for starting growth in both the mono-wavelength cases, combining the wavelengths drastically increases the probability of growth to 0.50. This result directly suggests a coupling effect between the two wavelengths occurring at the beginning of damage growth, as we have previously observed on the exit surface of a plane sample in Ref.^19^. This coupling may originate from the heating of the plasma (more likely produced in the UV) preferentially by the IR pulse according to Drude model^37^. Such processes have already been associated to similar multi-wavelength configurations in a wide variety of materials^38–40^. Another remarkable feature is that the coupling between the wavelengths is more efficient on the grating. Indeed, by comparing the results obtained at ${F}_{3{\rm{\omega }}}\approx 7$ J/cm^2^, for ${F}_{1{\rm{\omega }}}=0$ and ${F}_{1{\rm{\omega }}}=12.7$ J/cm^2^, the probability of growth jumps from ~0 to 0.5 on the grating, while it jumps from ~0 to 0.25 on a plane sample as previously established in Ref.^19^. As discussed in the previous section, this result is very consistent with the enhancement of the two optical electric fields induced by the pillar structure as well as with the additional defects on the grating due to the manufacturing process.

The experimental data are compared in Fig. 5 to the sigmoid curves reading:

5 $$P({F}_{1\omega },{F}_{3\omega })=1-\frac{1}{1+{(\frac{{F}_{1\omega }}{{F}_{1\omega }^{(50 \% )}})}^{p}+{(\frac{{F}_{3\omega }}{{F}_{3\omega }^{(50 \% )}-0.2{F}_{1\omega }})}^{p}}$$

where, ${F}_{1\omega }^{(50 \% )}$ and ${F}_{3\omega }^{(50 \% )}$ is the fluence for which the probability of growth is equal to 50% at 1ω and 3ω (see Table 1), respectively, and *p* = 10 is the exponent obtained in both mono-wavelength configurations. The expression in Eq. (5) has been selected for automatically fitting the mono-wavelength cases, which can be retrieved by setting ${F}_{1{\rm{\omega }}}=0$ or ${F}_{3{\rm{\omega }}}=0$. The term $0.2{F}_{1{\rm{\omega }}}$ has been introduced to account for the previously discussed shift found towards the lowest ${F}_{3{\rm{\omega }}}$ values in Fig. 5. Thereby, Eq. (2) reproduces fairly well the experimental trends and allows us to quantify the coupling between the two wavelengths.

**Figure 6:**
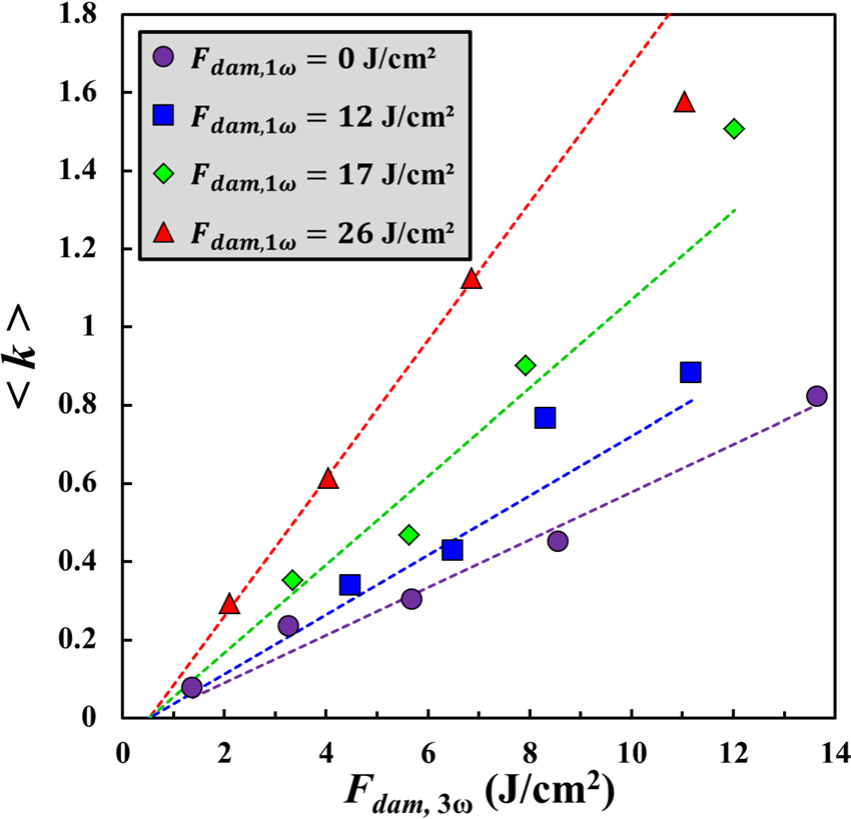
Evolution of growth coefficient <*k*> on the exit surface of the grating as a function of the corrected fluence at 3ω ${F}_{dam,3\omega }$ for several corrected fluences at 1ω ${F}_{dam,1\omega }$. The experimental multi-wavelength data are compared to the parameterizations issued from Eq. (6) at the same fluences at 1ω (dashed curves in the same colours) with the parameters reported in Table 2. The results obtained at 3ω only (purple circles and dashed line) are issued from Fig. 3.

Finally, we study the influence the combination of wavelengths on the damage growth phenomenon on the grating on a pulse-to-pulse basis. The growth coefficient $\langle k\rangle$ is thus displayed in Fig. 6 as a function of the corrected fluence at 3ω ${F}_{dam,3{\rm{\omega }}}$, for several corrected fluences at 1ω ${F}_{dam,1{\rm{\omega }}}$. For every ${F}_{dam,1{\rm{\omega }}}$ value, a linear increase in $\langle k\rangle$ with respect to ${F}_{dam,3{\rm{\omega }}}$ is found. The striking feature in Fig. 6 is that adding a 1ω flux directly impacts the rate of increase *C* in the growth coefficient since the slope of the experimental data increases with ${F}_{dam,1\omega }$. This behaviour strongly differs from the one found on a plane surface for similar conditions, where adding a 1ω flux only decreases the threshold fluence for growth *F*_*th*_ without modifying *C*^19^. Concerning the grating, one can note in Fig. 6 that for all the added ${F}_{dam,1\omega }$ values, *F*_*th*_ is the previously determined one at 3ω only (0.5 J/cm^2^).

In order to quantify the increase in slope in the multi-wavelength configuration, the experimental data are compared to the following expression:

6 $$\langle k({F}_{1\omega },{F}_{3\omega })\rangle =\alpha \langle k({F}_{1\omega })\rangle \langle k({F}_{3\omega })\rangle $$

where $\langle k({F}_{1\omega })\rangle ={C}_{1\omega }({F}_{dam,1\omega }-{F}_{th,1\omega })$, and $\langle k({F}_{3\omega })\rangle ={C}_{3\omega }({F}_{dam,3\omega }-{F}_{th,3\omega })$ are the average growth coefficients in the mono-wavelength configurations with ${C}_{1\omega }$, ${C}_{3\omega }$, ${F}_{dam,1\omega }$, and ${F}_{dam,3\omega }$ reported in Table 2, and the coefficient $\alpha =2.4$ is a factor determined after optimization which accounts for the measured change in slope. Consequently, the parameterization given by Eq. (6) reproduces fairly well the experimental trends, and directly suggests that the rate of increase in the growth coefficient is a linear function of ${F}_{dam,1\omega }$. Thereby, the multi-wavelength growth behaviour on the grating can be simply predicted by multiplying ${C}_{3\omega }$ by $\alpha \langle k({F}_{1\omega })\rangle$. As previously discussed, the mechanisms that may occur in the experiments in Fig. 6 is a plasma production preferentially at 3ω in the vicinity of the subsurface cracks of the damage, followed by a heating of the electrons more efficient at 1ω. The energy transfer from the electrons to the lattice is thereby more important in the multi-wavelength case than in the mono-wavelength ones, leading to a more significant expansion of the damage site.

## Conclusion

In summary, we have shown that LID growth phenomenon depends on the initial damage morphology. This has led us to carry out growth experiments on damage sites with similar morphology and size for sake of repeatability. The growth behaviour has then been characterized in the mono-wavelength configurations by determining the probability of growth as well as the growth coefficient. By comparison with a plane fused silica sample, the LID growth on the grating is significantly more important, due to the periodic surface structure. This is consistent with the local enhancement of the optical electric field intensity in the vicinity of the damage induced by the grating geometry, as confirmed by FDTD calculations. The determination of the growth behaviour in the mono-wavelength configurations has finally enabled us to quantify a coupling between the pulses at 1ω and 3ω in the multi-wavelength one, which could originate from the heating of the plasma (more likely produced in the UV) preferentially by the IR pulse. The complete set of results should find plenty of applications for high-energy laser facilities as well as for the fundamental comprehension of the damage growth phenomenon in the nanosecond regime.

## Materials and Methods

### Tested samples

The samples are 10 mm thick synthetic fused silica superpolished by SESO company. The grating has additionally been written with a holographic process described in Ref.^25^, and its surface geometry has been measured prior to damage experiments thanks to Scanning Electron Microscopy (SEM). The damage growth tests have been performed at an angle of incidence of 25°, and the sample is mounted on motor-driven stage. The tested damage sites are illuminated with white light and visualized by means of a long-focal microscope associated with a CCD camera for measuring their area before and after each irradiation. In order to avoid effects of a previously tested damage site on a new one, the sites are spatially separated by 3 mm. Finally, *post*-*mortem* inspections of several damage sites have been carried out thanks to Nomarski Interference Contrast (NIC) microscopy.

### Laser-induced damage experiments

The laser facility described in Ref.^40^ has been employed for the initiation and the growth of damage sites on the exit surface of the samples. Briefly, it consists in a tripled Nd:YAG giving access to laser pulses at 1064 nm (1ω) and 355 nm (3ω) separately (mono-wavelength configurations) or simultaneously (multi-wavelength configuration), at a repetition rate of 10 Hz. The laser cavity shows multiple longitudinal modes (MLM) and the pulse duration is ~6.5 ns at 1ω, and ~5.5 ns at 3ω. The optical path length is the same for the two beams, ensuring their synchronization. The two beams are separated and recombined by means of dichroic mirrors, so they are collinear after focusing with lenses whose focal length are approximately 4 m. The delivered energy of each beam is adjusted by combining a half-wave plate and a polarizer. At the focus the two beams are Gaussian-shaped and the beam diameter (measured at 1/e) is about 700 µm at 1ω, and 500 µm at 3ω. Since the depth of focus is much higher than the thickness of the sample, the diameter of each beam is considered as constant along its propagation through the sample. The maximum fluence that may be reached is 130 J/cm^2^ at 1ω, and 75 J/cm^2^ at 3ω.

### Laser metrology

In order to determine the fluence at both wavelengths, the energy is calibrated before the experiments. This calibration procedure consists in measuring the energy of the beam at the focus (*E*_*foc*_) and the energy of the transmitted light of a dielectric mirror (*E*_*mes*_) simultaneously with two energy meters. The linearity between *E*_*mes*_ and *E*_*foc*_ enables us to evaluate the energy *E*_*foc*_ of every pulse during the growth sequence by simply recording *E*_*mes*_. The fluence of each pulse is then retrieved by dividing *E*_*foc*_ by the equivalent area of the beam at the focus *S*_*eq*_. This area is measured at 1/e for each pulse by means of CCD cameras positioned so that the distance between the focusing lens and the sample is identical as the one between the lens and the camera. Finally, in order to account for the angle of incidence of 25° between the sample and the optical axis, the obtained fluence value is multiplied by $\cos (25^\circ )\approx 0.91$.

### Data processing

The probability of growth is obtained by calculating the ratio between the number of growing damage sites and the total number of tested sites (between 5 and 15) at a given laser fluence. A damage site is considered as non-growing if the ratio between its final and initial size is <2 after 200 laser pulses. Concerning growing damage sites, the growth coefficient is calculated after every pulse as the logarithmic ratio between the damage areas after and before the pulse. The damage area is retrieved with ImageJ software. This growth coefficient is associated with a corrected fluence value accounting for the size of the damage site, as reported in Ref.^19^. Finally, the growth sequence is stopped when the damage size exceeds the one of the incoming beam in the mono-wavelength cases, and the one of the beam at 3ω in the multi-wavelength case since this latter is smaller than at the one at 1ω.

### Numerical simulations

A Finite-Difference Time-Domain (FDTD) solver^41^ was employed to perform the calculations of the enhancement of the optical electric field intensity. The time-dependent Maxwell’s equations are solved for an incident plane wave with a linear polarization. The angle between the sample and the laser beam polarization is 25°, as in the experiments.

### Data availability

The datasets generated during and/or analysed during the current study are available from the corresponding author on reasonable request.
